# Association Between Vitamin D Levels and Glycemic Control Among Adult Diabetic Patients in Riyadh, Saudi Arabia

**DOI:** 10.7759/cureus.25919

**Published:** 2022-06-13

**Authors:** Saleh Abubaker, Abdulrahman Albasseet, Kossay A El-abd, Asim A Alandijani, Yaser A Alendijani, Abdullah Alkhenizan

**Affiliations:** 1 Family Medicine, King Faisal Specialist Hospital and Research Centre, Riyadh, SAU; 2 Research, King Faisal Specialist Hospital and Research Centre, Riyadh, SAU; 3 College of Medicine, King Abdulaziz University, Jeddah, SAU; 4 Family Medicine and Polyclinics, King Abdulaziz University, Riyadh, SAU

**Keywords:** serum 25-hydroxyvitamin d, vitamin d, glycemic control, diabetic, diabetes

## Abstract

Background*:* Diabetes is one of the most common diseases worldwide. It can cause serious complications, such as cardiovascular events, end-stage renal disease, and blindness if not controlled. Vitamin D is believed to play an essential role in glucose metabolism and insulin resistance. However, few studies have been conducted in Saudi Arabia to confirm or reject this hypothesis. Thus, this study explored the relationship between vitamin D levels and glycemic control in a Saudi diabetic population.

Materials and methods: This is a retrospective cohort study including all adults 18 years of age or older diagnosed with diabetes who underwent at least five years of regular follow-up at the family medicine clinic at the King Faisal Specialist Hospital (KFSH) from January 2015 to January 2021. Data were obtained from the patients’ medical records and included detailed histories, physical examination records, and laboratory findings. Participants were divided into vitamin D deficiency and vitamin D sufficiency groups based on vitamin D levels.

Results: A total of 370 patients with type 2 diabetes mellitus were enrolled in the study. The majority of the patients (60%) were over 65 years of age. The mean serum 25(OH) vitamin D level of the participants was 62.75 ± 22.79 nmol/L. There was a significant association between glycemic control and vitamin D levels (p < 0.001). The mean level of vitamin D was higher in the good glycemic control group (70.96 ±22.66) than in the poor glycemic control group (54.81 ±19.98). A total of 13.74% (25) of the good glycemic control group had vitamin D levels < 50 nmol/L, while 52.13% (98) of the poor glycemic control had vitamin D levels < 50 nmol/L. Patients with poor glycemic control were 2.4 times more likely to have low vitamin D levels than patients in the well-controlled glycemic group.

Conclusion: Based on the study results, serum vitamin D has a significant inverse relationship with HbA1c levels among diabetics. This finding highlights the need for routine screening of vitamin D status in all patients with diabetes and early treatment for those found to be deficient.

## Introduction

Diabetes mellitus (DM) is a major global health concern, and one of the four noncommunicable diseases targeted for action by world-leading governments [1]. According to the International Diabetes Federation (IDF), there are currently 363 million people with diabetes worldwide and this number is expected to increase to 700 million by the year 2045 [2]. Saudi Arabia is among the most affected countries by DM, with local reports suggesting the overall prevalence of the disease to be 23.7% [3].

If not adequately controlled, DM can lead to serious complications. For example, it increases the risk of a cardiovascular event by two to three times [1]. Furthermore, it has been reported that at least 80% of end-stage renal disease (ESRD) is caused by either diabetes or hypertension [1]. Diabetic retinopathy is one of the most common causes of blindness, accounting for an estimated 2.9% of all cases worldwide [1]. Therefore, DM imposes a significant economic burden on the global health system. According to the IDF, the total diabetes-related global health expenditure is around USD 760 billion per year and the economic impact is expected to grow to USD 825 billion per year by 2030 [2]. Saudi Arabia is no exception to this trend, as it has been estimated that the expenses related to diabetic patients represent around 14% of the overall health expenditure in the country [4].

Vitamin D plays an essential part in glucose metabolism. Including improving insulin exocytosis, stimulating insulin receptors, improving the uptake of glucose by peripheral tissues, and improving insulin resistance [5-7]. Hence, vitamin D plays a pivotal role in maintaining normal glucose levels. However, few studies have been conducted on this subject in Saudi Arabia to the best of our knowledge. Accordingly, this study explores the relation between vitamin D levels and glycemic control in a Saudi diabetic population.

## Materials and methods

This was a retrospective cohort study, which was conducted following the recommendations of the Declaration of Helsinki and the policies of the Research Advisory Committee (RAC) at King Faisal Specialist Hospital (KFSH) and Research Centre, as well as per the laws of the Kingdom of Saudi Arabia (RAC# 2211006). In this study, we included all adults 18 years of age or older diagnosed with diabetes who underwent regular follow-up for at least five years at the family medicine clinic at KFSH from January 2015 to January 2021. Data were obtained from the patients’ medical records and included detailed histories, physical examination records, and laboratory findings (age, gender, nationality, body mass index (BMI), fasting blood glucose (FBG), hemoglobin A1c (HbA1c), and 25-hydroxyvitamin D (25(OH)D) levels).

HbA1c and fasting glucose levels were measured using the ion-exchange high-performance liquid chromatography technique, while 25(OH)D levels were measured using an enzyme-linked immunosorbent assay (ELISA).

Laboratory values (FBG, HbA1c, and 25(OH)D levels) were based on measurement at regular intervals (3-6 months) and the average of all readings in the five years of follow-up. The last follow-up visit recorded other variables (age, gender, nationality, and BMI). Patients with less than five different laboratory readings and those with chronic liver and kidney disorders, malignancies, and endocrinology disorders such as hypothyroidism, hyperthyroidism, and hyperparathyroidism were excluded from the study.

Patients were defined as having diabetes if they were coded with “E10-E14,” which stands for diagnosis of “Diabetes Mellitus” based on the International Classification of Diagnosis (ICD) version 10 code, or if they were diagnosed with new-onset diabetes according to the diagnostic criteria of the American Diabetes Association (ADA), which are shown in Table 1 [8].

**Table 1:** Diagnostic criteria for diabetes according to American Diabetes Association (ADA) HbA1c: hemoglobin A1C; FBG: fasting blood glucose; 2-h OGTT: 2-h oral glucose tolerance test; RBG: random blood glucose

Diagnosis	Test			
Diabetes	HbA1c (%)	FBG (mg/dL)	2-h OGTT (mg/dL)	RBG test (mg/dL)
	6.5 or above	126 or above	200 or above	200 or above

The participants were divided into two classes based on vitamin D levels: those with serum 25(OH)D levels less than 50 nmol/L (20 ng/mL) were classified as the vitamin D deficiency group. In contrast, those with serum 25(OH)D levels of 50 nmol/L (20 ng/mL) or more were classified as the vitamin D sufficiency group [9]. Similarly, the patients were divided into two groups based on HbA1c levels: good glycemic control and poor glycemic control groups. This classification was based on the ADA’s Standards of Care recommendations [10]. According to ADA guidelines, the participants were considered to have controlled diabetes if their HbA1c levels were less than 7%.

Statistical analysis

The statistical analysis was performed using the statistical software package SPSS (version 20.0; IBM, Armonk, New York, United States). Descriptive statistics for the continuous variables were reported as means and standard deviations, and categorical variables were summarized as frequencies and percentages. Continuous variables were analyzed using an independent t-test and an analysis of variance (ANOVA). Categorical variables were compared using the Chi-squared test. Univariate and multivariate regressions were conducted to evaluate the risk of developing poor glycemic control and the level of statistical significance was set at p < 0.05.

## Results

A total of 370 patients diagnosed with type 2 DM were enrolled in this study. The mean age of these patients was 65 ±10.5 years. The majority of the patients (60%) were considered to be geriatric patients (i.e., over 65 years old). Regarding gender, the study population was approximately distributed equally, with 50.8% females (188) and 49.2% males (182). The large majority of patients, 84.1% (311), were Saudi. Less than 1% of the patients were underweight, 29.5% were overweight, and 56.2% were obese, with a mean total BMI of 30.9 ± 5.9 kg/m^2^. Moreover, the mean serum 25(OH) vitamin D level for all the participants was 62.75 ± 22.79 nmol/L. Table 2 summarizes the demographic characteristics of the participants.

**Table 2:** Demographic characteristics of all participants N: number of cases; SD: standard deviation; BMI: body mass index; HbA1c: hemoglobin A1c; FBG: fasting blood glucose; mmHg: millimeters of mercury

Variables	N/mean	%/SD
Age	65.01	10.53
Less than 65	148.00	40.00
65 and above	222.00	60.00
Gender		
Female	188.00	50.80
Male	182.00	49.20
Nationality		
Saudi	311.00	84.10
Non-Saudi	59.00	15.90
BMI	30.94	5.86
Underweight	3.00	0.81
Normal	50.00	13.51
Overweight	109.00	29.46
Obese	208.00	56.22
Vitamin D level	62.75	22.79
≥ 50 nmol/L	247.00	66.80
< 50 nmol/L	123.00	33.20
HgA1c level	7.32	2.76
Good glycemic control	182.00	49.20
Poor glycemic control	188.00	50.80
FBG	7.88	3.74
Type of diabetic medication		
Non-insulin	232.00	62.70
Insulin	61.00	16.50
Both	77.00	20.80
Route of administration		
Oral	179.00	48.38
Injectable	56.00	15.14
Both	135.00	36.49
Number of years lived with diabetes	6.95	1.78
Systolic blood pressure	132.98	15.26
< 140 mmHg	248.00	67.03
≥ 140 mmHg	122.00	32.97
Diastolic blood pressure	76.33	9.06
< 90 mmHg	344.00	92.97
≥ 90 mmHg	26.00	7.03

Although no significant association was found between the level of glycemic control and personal characteristics, including age, gender, nationality, or BMI (p > 0.05), there was a significant association between glycemic control and vitamin D levels (p < 0.001). The mean vitamin D level was higher in the good glycemic control group (70.96 ± 22.66) than in the poor glycemic group (54.81 ± 19.98). In the good glycemic control group, 13.74% (25) of the patients had vitamin D levels < 50 nmol/L, while in the poor glycemic control group, 52.13% (98) of the patients had vitamin D levels < 50 nmol/L. FBG, systolic blood pressure, and type of diabetic medication also had a significant association with the level of glycemic control (p < 0.001). Table 3 summarizes the demographic characteristics of the good and poor glycemic control groups.

**Table 3:** Demographic characteristics of controlled diabetes and uncontrolled diabetes groups BMI: body mass index; FBG: fasting blood glucose; mmHg: millimeters of mercury

Variables	Good glycemic control	Poor glycemic control	p-value
Age	65.42 +/- 10.55	64.62 +/- 10.52	0.468
Less than 65	76	72	0.497
65 and above	106	116	
Gender			0.347
Female	97	91	
Male	85	97	
Nationality			0.157
Saudi	148	163	
Non-Saudi	34	25	
BMI	31.01 +/- 5.86	30.88 +/- 5.88	0.840
Underweight	1	2	0.811
Normal	23	27	
Overweight	57	52	
Obese	101	107	
Vitamin D level	70.96 +/- 22.66	54.81 +/- 19.98	0.000
< 50 nmol/L	25	98	0.000
≥ 50 nmol/L	157	90	
FBG	6.27 +/- 0.972	9.45 +/- 4.662	0.000
Type of diabetic medication			0.000
Non-insulin	173	59	
Insulin	3	58	
Both	6	71	
Route of administration			0.000
Oral	133	46	
Injectable	10	46	
Both	39	96	
Number of years lived with diabetes	6.68 +/- 1.73	7.22 +/- 1.79	0.003
Systolic blood pressure	129.23 +/- 14.30	136.61 +/- 15.30	0.000
< 140 mmHg	140	108	0.000
≥ 140 mmHg	42	80	
Diastolic blood pressure	75.45 +/- 8.70	77.18 +/- 9.34	0.066
< 90 mmHg	172	172	0.256
≥ 90 mmHg	10	16	

According to the multivariate logistic regression, age, gender, and BMI were not significantly associated with glycemic control. However, after adjusting for other risk factors, the results indicated a significant association between vitamin D levels and glycemic control. Univariate and multivariate logistic regressions were performed to evaluate the impact of confounding variables in the good and poor glycemic control groups. After adjusting for other risk factors, patients with poor glycemic control were 2.4 times more likely to have low vitamin D levels than patients in the well-controlled glycemic group (Table 4).

**Table 4:** Univariate and multivariate logistic regression BMI: body mass index; FBG: fasting blood glucose

Variables	Crude OR	Lower CI	Upper CI	p-value	Adjusted OR	Lower CI	Upper CI	p-value
Age	0.993	0.974	1.012	0.467	1.000	0.965	1.036	0.987
Gender (Male/Female)	1.216	0.809	1.830	0.347	1.180	0.562	2.477	0.662
BMI	0.996	0.962	1.032	0.838	0.990	0.932	1.052	0.754
Vitamin D (<50/≥50)	6.838	4.107	11.387	0.000	2.374	1.078	5.229	0.032
FBG	3.878	2.896	5.192	0.000	3.093	2.198	4.353	0.000
Type of diabetic medication								
Non-insulin	Reference			0.000	Reference			0.000
Insulin	56.689	17.117	187.747	0.000	17.516	3.345	91.730	0.001
Both	34.698	14.333	83.998	0.000	23.756	8.332	67.737	0.000

## Discussion

This study aimed to evaluate whether a relationship between vitamin D levels and glycemic control among diabetic patients exists. The findings support the hypothesis that these variables have a relationship, as a significant association was observed. These findings were replicated after adjusting for other risk factors. In addition, FBG, systolic blood pressure, and type of diabetic medication were found to be significantly associated with vitamin D levels.

The relationship between vitamin D levels and glycemic control has been widely investigated in the literature, with contradicting results. It has been suggested that vitamin D plays a favorable role in glucose homeostasis and glycemic metabolism; however, there is little evidence to support this claim [11]. A 2015 study investigated the impact of vitamin D levels on glycemic control in 128 patients with type 2 diabetes. No significant association was found between vitamin D levels and glycemic control across the cohort, despite a high level of vitamin D deficiency [12]. Conversely, a 2013 study reported that vitamin D levels were lower in patients with type 2 diabetes than in the control group. The study investigated vitamin D3 levels and their relationship with glycemic control in 120 patients with type 2 diabetes. An inverse relationship was found between vitamin D3 levels and glycosylated hemoglobin levels [13].

The findings reported in the current study are supported by the positive outcomes observed in patients given vitamin D supplementation. A 2019 meta-analysis reported that short-term vitamin D interventions in patients with type 2 diabetes improve HbA1c levels, and insulin sensitivity, thereby improving glycemic control [14]. These results were mirrored in an additional review, which found that vitamin D supplementation effectively reduced insulin resistance and yielded highly beneficial outcomes when given in large doses for a short period of time [15]. Our findings are further supported by a 2014 randomized controlled trial that evaluated the impact of vitamin D supplementation on glucose control and insulin resistance in 28 patients with type 2 diabetes. Participants received 4000 IU of vitamin D over two months [16]. The results demonstrated that vitamin D decreased blood glucose levels and enhanced insulin sensitivity in type 2 diabetics.

Several limitations must be considered when interpreting the findings of this study. The retrospective nature of the research limits the quality of the evidence provided, and the risk of bias is substantially greater than if a prospective approach had been followed. This design introduces selection bias and misclassification bias within the findings. In addition, the data were collected from a single center, the KFSH. Thus, the results could be specific to this center and surrounding geographical areas, thus compromising external validity. To reduce these limitations, it is recommended that future research be conducted at multiple centers spanning several geographical locations. Data should also be collected prospectively and over an extended period. Future research should investigate the relationship between vitamin D levels and FBG, systolic blood pressure, and type of diabetic medication.

## Conclusions

Based on the results of this study, serum vitamin D levels in diabetics show a significant inverse relationship to HbA1c levels. This highlights the need for routine screening of vitamin D status in all patients with diabetes and early treatment for those deficient. Such treatment would help achieve better glycemic control and prevent the development or worsening of diabetes‐related complications. Additional studies are needed to address the limitations identified above and produce results with greater external validity.
